# Association of the Serum Folate and Total Calcium and Magnesium Levels Before Ovarian Stimulation With Outcomes of Fresh *In Vitro* Fertilization Cycles in Normogonadotropic Women

**DOI:** 10.3389/fendo.2022.732731

**Published:** 2022-02-11

**Authors:** Mikhail Polzikov, Dmitry Blinov, Zarema Barakhoeva, Lyudmila Vovk, Yulia Fetisova, Maria Ovchinnikova, Marina Tischenko, Irina Zorina, Vasily Yurasov, Tatyana Ushakova, Oleg Sergeyev

**Affiliations:** ^1^ IVFarma LLC, Moscow, Russia; ^2^ Institute for Preventive and Social Medicine, Moscow, Russia; ^3^ Lapino Clinical Hospital, MD Medical Group, Moscow, Russia; ^4^ In Vitro Fertilization (IVF) Department, “AltraVita” Human Reproduction Clinic, Moscow, Russia; ^5^ Perinatal Medical Center, MD Medical Group, Moscow, Russia; ^6^ In Vitro Fertilization (IVF) Department, “NovaClinic” Center of Reproductive Medicine and Genetics, Moscow, Russia; ^7^ Laboratory of Chromatographic Systems LLC, Moscow, Russia; ^8^ Belozersky Institute of Physico-Chemical Biology, Lomonosov Moscow State University, Moscow, Russia

**Keywords:** folate, folic acid, ART, *in vitro* fertilization, nutrition supplements, ovarian stimulation, calcium, magnesium

## Abstract

**Background:**

Women of reproductive age are recommended to consume folic acid and other supplements before conception and during pregnancy. We aimed to investigate the association of the serum folate and total magnesium (Mg) and calcium (Ca) levels before ovarian stimulation with the outcomes of assisted reproductive technology (ART) in normogonadotropic women.

**Methods:**

We used a subanalysis of data obtained from a multicentre, randomized prospective study (NCT03088137). A total of 110 normogonadotropic, non-advanced aged, non-obese women with tubal and/or male infertility factors were enrolled for the single fresh ovarian stimulation GnRH antagonist cycle. The main outcome measures were the total oocyte yield, mature oocytes, fertilization rate, biochemical, clinical pregnancy, and live birth. Multivariable generalized linear models adjusted for covariates were used with a Poisson distribution and the log link function for adjusted oocyte counts, and a binomial distribution and the log link function were used for adjusted clinical ART outcomes.

**Results:**

The medians (interquartile range (IQR)) were as follows: baseline serum folate, 20.55 ng/ml (10.8, 32.9); Mg, 19.4 mg/L (18.7, 20.7); Ca, 94 mg/L (91.2, 96.4); and Ca/Mg ratio, 4.78 (4.55, 5.02). Women with higher serum folate concentrations (Q4≥33.0 ng/ml) had significantly lower total numbers of oocytes retrieved (adjusted mean (95% CI) 9.2 (7.6-11.3) vs 12.9 (10.9-15.4, p-trend=0.006)) and lower odds ratios (ORs) (95% CI) of 0.12 (0.02, 0.79) for clinical pregnancy and 0.10 (0.01, 0.70) for live birth compared with women in the lowest quartile (<10.8 ng/ml), all p-trend<0.001. Women in the highest Ca/Mg ratio quartile (≥5.02) had ORs (95% CI) of 6.58 (1.31, 33.04) for biochemical pregnancy, 4.85 (1.02, 23.08) for clinical pregnancy and 4.07 (0.83, 19.9) for the live birth rate compared with women in the lowest quartile (<4.55), all p-trend<0.001.

**Conclusions:**

Using multivariable models, we suggested that a baseline elevated serum folate level (≥33.0 ng/ml) and a lower Ca/Mg ratio were associated with worse ART outcomes in normogonadotropic women. Our findings might be useful for choosing safe dosages of folate, calcium, magnesium and complex supplementation for both fertile women and women undergoing infertility treatment. Further preconception large-scale studies with known micro- and macronutrient statuses of both parents and serum folate, Ca, Mg, and hormone levels, are needed.

## Introduction

Micronutrient deficiencies before and during pregnancy affect reproductive health and increase the risks of adverse pregnancy outcomes for mothers and children. The International Federation of Gynaecology and Obstetrics (FIGO) recommends a varied and healthy diet and provision of supplements or fortified foods throughout pregnancy and the periconceptional period aiming to compensate for possible deficiencies and increased nutrient requirements (1). For most women who are planning to become pregnant, a wide range of over-the-counter complex vitamins, mineral preparations, amino acids and botanicals are recommended for use before and during pregnancy (1–3); however, personal requirements based on individual levels in body and diet characteristics are not considered. Overconsumption of micronutrients can affect pregnancy outcomes and children’s health, including an increased risk of early fetal death (4) and altered psychomotor development (5).

Folic acid or folate (natural form) is an essential micronutrient for many physiological processes, and its nutritional demands increase during pregnancy (1, 3, 6). The recommended dosage of folic acid from supplementation in addition to dietary folate intake is 400 μg/day and is based on the prevention of neural tube defects (1, 3, 6). However, more than 57% of women did not reach the recommended dosage of 400 μg/day during pregnancy, 25% took more than 1000 μg/day and 3.5% consumed >5000 μg/day of folic acid supplements (5).

Calcium (Ca) and magnesium (Mg) are other important micronutrients for pregnant women (3). A high incidence of Mg deficiency was demonstrated in a Russian observational study among pregnant women and women with hormone-dependent diseases (7, 8). It was shown that reductions in serum levels of calcium and magnesium during pregnancy might be possible contributors to the etiology of preeclampsia and that supplementation of these elements to the diet may be of value to prevent preeclampsia (9). In other studies, hypocalcemia correlated with severe preeclampsia, while magnesium supplementation did not reduce the incidence of preeclampsia in pregnant women (10–12). Muneyvirci-Delale et al. reported the recurring cycling of ionized Mg and cyclic alterations in the ionized Ca to Mg ratio in the serum of healthy women over the menstrual cycle, which was accompanied by changes in the levels of the sex steroids, estradiol, progesterone and testosterone (13).

Adequate folate status has also been broadly investigated before and during assisted reproductive technology (ART), and significant differences in the prevalence of folic acid intake among countries have been revealed (14–16). The results of studies investigating the folate status, supplementation before and during ART and ART outcomes are rather controversial. On the one hand, a higher serum concentration of folate before ART treatment was associated with higher live birth rates among American women using different treatment protocols (17). Conversely, women with unexplained infertility had significantly higher median plasma folate concentrations than fertile women, and a good folate status did not have a positive effect on pregnancy outcome following ovarian stimulation (OS) (18). Moreover, higher concentrations of serum folate can modulate the ovarian response to gonadotropin during OS with GnRH-agonist by influencing estradiol levels, which is associated with a lower estradiol response and lower follicle number in the folic acid users group in comparison with nonusers (19). It was shown that a diet high in synthetic folate can be associated with increased endogenous progesterone levels and a lower risk of sporadic anovulation (20).

Using our data obtained from a therapeutic equivalence study of follitropin alpha biosimilar (21), we aimed to investigate the associations of serum folate, vitamin B12, magnesium and calcium levels before ovarian stimulation with the outcomes of ART in a single GnRH antagonist (GnRH-ant) protocol in normogonadotropic women.

## Materials and Methods

This was a subanalysis of data obtained from a multicentre, randomized, embryologist-blinded, parallel-group, therapeutic equivalence study of follitropin alpha biosimilar (ClinicalTrials.gov Identifier: NCT03088137) (21). The major results of this study argue in favour of both investigated follitropins being therapeutically equivalent; therefore, the clinical data obtained in this study for randomized patients in both compared groups can be combined and considered for further analysis in the current substudy.

### Ethics Approval and Consent to Participate

The study protocol and informed consent were approved by the Russian Ministry of Health (RCT 754 dated 26.10.16) and the Independent Interdisciplinary Ethics Committee for Ethical Review of Clinical Studies established by Moscow State University of Medicine and Dentistry, Russian Academy of Medical Sciences, Society for Pharmacoeconomics and Outcomes Research, League to support clinical studies and the Scientific Center for Medical Information ‘‘Universimed’’, www.ethicuni.ru (protocol №17 dated 28.10.2016). All participants provided written informed consent prior to enrolment, and the first subject was enrolled on 08.02.2017.

### Study Population

**Figure 1:**
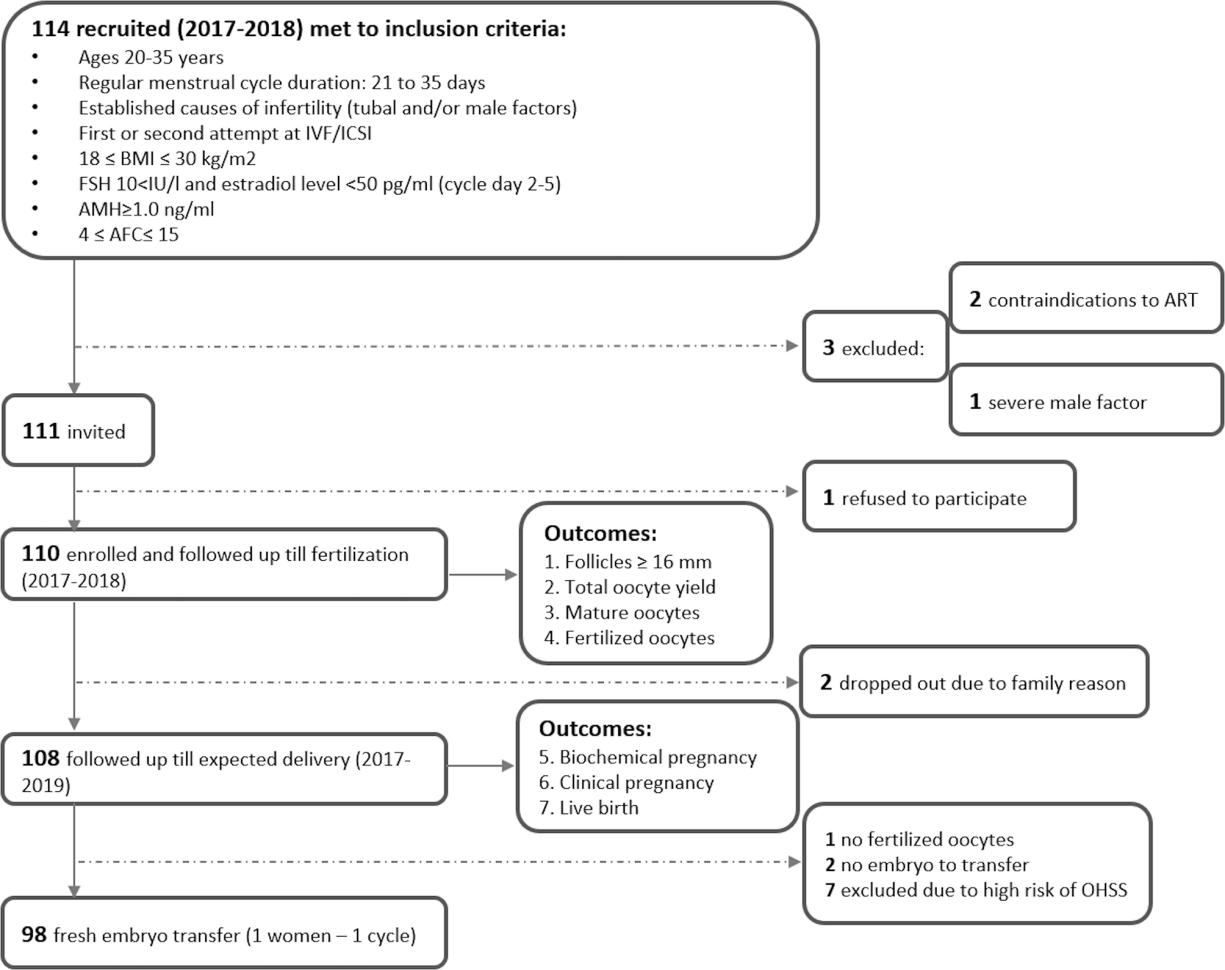
Flowchart of recruitment and follow up study subjects.

The flowchart of study recruitment and follow-up is presented in **Figure 1**. In 2017-2018, 114 women were enrolled in the parental study of the therapeutic equivalence of follitropin alpha in three IVF centres in Moscow, Russia (“AltraVita” Human Reproduction Clinic, Perinatal Medical Center, and Lapino Clinical Hospital) using the following inclusion criteria: women aged 20-35 with a regular menstrual cycle; tubal and/or male causes of infertility factors; first or second attempt at IVF/ICSI; 18 ≤ BMI ≤ 30 kg/m^2^; FSH level <10 IU/l, estradiol level <50 pg/ml and AMH level ≥1.0 ng/ml at cycle days 2-5; 4 ≤ antral follicular count (AFC) ≤ 15; no pathology of the endometrium; and no sexually transmitted infections (21). Three women were excluded due to contradictions to ART (n=2) and a severe male factor (sperm concentration <2 10^6^/ml, n=1), and 1 woman refused. A total of 110 women were enrolled and followed up within the ART protocol prior to outcomes by 14 obstetricians who had standardization training before the study. The serum of all 110 women from parental study NCT03088137 was analyzed for micronutrients.

### Clinical Procedures

**Figure 2:**
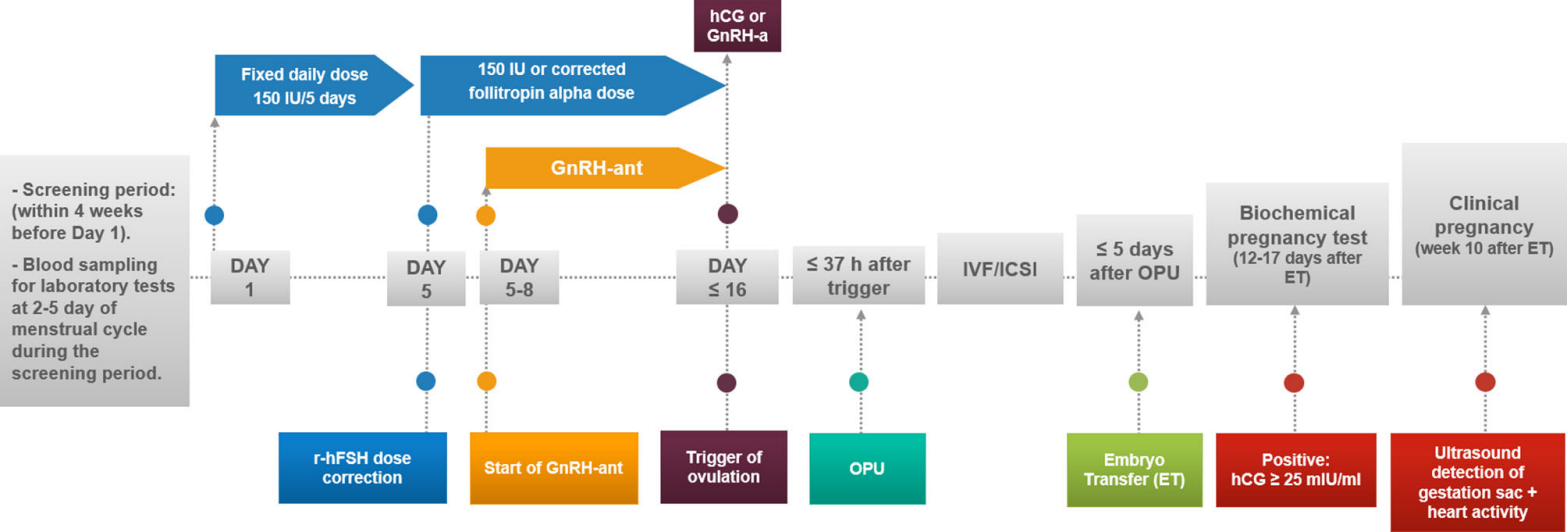
Time-line of biospecimen collection, clinical procedures and assessment of ART outcomes.

No tight restrictions for folic acid or other food supplements/vitamins were applied before, during or after OS, with the usual recommendation of taking 800 μg/day folic acid. Participants underwent a single OS follicular-phase GnRH antagonist protocol in one fresh cycle. Starting on days 2-3 of the menstrual cycle after the screening period, a fixed daily subcutaneous (SC) dose of 150 IU of follitropin alpha (Gonal-f^®^, Merck, Germany or Primapur^®^, IVFarma, Russia) was administered daily for 5 days, with possible correction after day 5 based on ultrasound examinations (**Figure 2**) (21). A GnRH-ant ganirelix acetate (Orgalutran^®^, MSD, Netherlands) at 0.25 mg was added daily starting when the leading follicle reached a mean diameter of 14 mm less than 37 hours after the intramuscular administration of either 5000-10 000 IU of hCG (Pregnyl^®^, MSD, Netherlands), n=92 (84%), or 0.2 mg of GnRH-a (GnRH agonist) (Decapeptyl^®^, Ferring Pharmaceuticals, Switzerland) in 18 (16%) women at risk for ovarian hyperstimulation syndrome (OHSS). The hCG trigger criteria were leading 2-3 follicles ˃ 18 mm; the GnRH-a criteria in women at risk of OHSS were growth of more than 15 follicles ˃ 14 mm on the day of trigger. Transvaginal oocyte retrieval was performed, followed by IVF or ICSI according to the centre’s standard procedures as well as luteal phase support. The transfer of a maximum of 2 embryos/blastocysts was allowed after oocyte retrieval with endometrial thicknesses not less than 8 mm.

### Biospecimen Collection and Assessment of Micronutrients and Hormones in the Serum

Blood samples were collected at 2-5 days of the menstrual cycle during the screening period within 4 weeks prior to day 1 and the administration of follitropin alpha (**Figure 2**). Fresh serum and whole blood were transported to three accredited clinical laboratories, INVITRO, CITILAB, and the laboratory of the Perinatal Medical Center, MD Medical Group, Moscow, Russia, for assessments of the blood count, biochemical parameters, including total protein, parameters of hemostasis and the following hormones: AMH; FSH, LH, estradiol, prolactin, total testosterone, cortisol, TSH, ACTH, and GH. Serum for micronutrient analysis [folate, B12, total magnesium (Mg) and calcium (Ca)] was stored below -70°C (22) and analyzed in one batch at “Chromolab”, Moscow, Russia. The serum folic acid and vitamin B12 levels were measured by an Architect i2000 microparticle chemiluminescent immunoassay analyser (Abbott, USA) with a LOD of 0.5 ng/ml and a LOQ of 1.5 ng/ml for folate and 125 pg/ml and 150 pg/ml for vitamin B12, respectively. Nine samples of the 110 (8%) were above the upper limit of detection for folate (49 ng/ml) and were treated as 49 ng/ml. The serum total Ca and total Mg concentrations were measured by atomic absorption spectrometry (AAS) using an AA-7000 spectrometer (Shimadzu, Japan) with a LOD of 3 mg/l and a LOQ of 5 mg/l for Ca and 1 mg/l and 4 mg/l for Mg, respectively. All samples were within the detection limits. The calcium/magnesium ratio (Ca/Mg ratio) was calculated using values measured in mg/l.

### Covariate Assessment

Fourteen trained obstetricians collected covariate information, including age, height, weight, calculated BMI, history of infertility (duration and causes, IVF/ICSI attempts), and reproductive and applied clinical parameters. The diet was not assessed before the ART cycle. Ten embryologists evaluated semen quality of male partners and embryo quality routinely in accordance with their own standards implemented in each clinic. Given no specific standardization training before the study for embryologists to assess semen and embryo quality and expected high between-observer variability, we did not use parameters of basic semen analysis as covariates and grades of embryo quality as outcomes in main models.

### Outcome Assessment

Seven consecutive outcomes of ART efficiency were evaluated. Early ART outcomes for 110 women included the number of follicles ≥ 16 mm on the day of hCG (or GnRH-a) administration; the number of oocytes retrieved; oocyte quality outcome (MII stage of maturity); and the number of fertilized oocytes (zygotes with two pronuclei, 2PN). Fertilization was not successful (the number of fertilized oocytes was zero) for one participant of 110, and for two, there was no embryo to transfer. For 7 women with a high risk of OHSS, embryo transfer was cancelled. Therefore, for those 10 women for whom embryos were not transferred for reasons potentially related to predictors (no embryo to transfer, high risk of OHSS), their subsequent outcomes were considered negative. Two participants dropped out of the study due to movement from clinics for family reasons and were excluded from the evaluation of late outcomes. Thus, the late clinical ART outcomes were estimated for 108 women and included biochemical pregnancy (on days 12-17 after embryo transfer with a positive test for beta hCG ≥ 25 mIU/ml), clinical pregnancy (defined as a gestational sac with fetal heart activity occurring at the 10^th^ week after embryo transfer), and the take-home baby rate as the final outcome (**Figure 1**).

### Statistical Methods

Spearman correlation analysis was used for the initial search of associations between the baseline levels of micronutrients and study outcomes. Since we found that the baseline B12 level was not associated with any ART outcomes, we did not focus on B12 in further analysis. Then, women were classified into quartiles based on the serum folate concentrations and Ca/Mg ratios. Descriptive statistics were calculated for demographic, reproductive, and laboratory characteristics according to these quartiles. Differences between the highest and lowest quartiles of investigated micronutrients were tested using the Kruskal-Wallis test for continuous variables and the chi-square test for categorical variables. Generalized linear models were used to evaluate the association between serum folate concentrations, Ca/Mg ratios and ART outcomes while accounting for within-person correlations in outcomes. A Poisson distribution and log-link function were specified for oocyte counts, and a binomial distribution and log-link function were specified for late clinical outcomes as recommended for observational studies of ART (23). Tests for trends across quartiles were conducted by using the *nptrend* command by STATA (24), and individual predicted values in each model were grouped by quartiles.

Confounding was evaluated by using prior knowledge and descriptive statistics from our cohort. The core model includes more than 30 covariates that can have an effect on the ART cycle. For each outcome, we fit a full multivariable model including all covariates as univariate p ≤ 0.20 for that outcome. The following covariates were considered for inclusion in models as factors: age (continuous), BMI (continuous), female and male factors of infertility (yes or no), infertility duration (continuous), number of previous ART attempts (0 or 1), type of follitropin alpha (Gonal-f^®^ or Primapur^®^), ovulation trigger (GnRH-a or hCG), duration of stimulation (continuous), FSH dose (continuous) and baseline serum concentrations of hormones (LH, FSH, AMH, estradiol, prolactin, and testosterone; continuous). Parameters of hemostasis (antithrombin and activated partial thromboplastin time (APTT); continuous) (25) were included, as well as red blood cell counts (RBC; continuous) because low RBC counts may indicate vitamin B12 and folate deficiency (3); and total protein level (continuous). The model for each outcome was reduced to a final model retaining covariates with p < 0.10. In these models, we included quartiles of the serum folate concentration and Ca/Mg ratio and evaluated the associations between the quartiles of the serum folate concentration, Ca/Mg ratio and each outcome. Since two oocyte outcomes, number of follicles ≥16 mm and retrieved oocytes, were highly correlated, we used only latest one for models. We conducted statistical analyses using Statistica version 12.0 (StatSoft, Inc., USA) and considered 2-sided significance levels of p<0.05 to be statistically significant. For oocyte counts, the results are presented as population marginal means adjusted for covariates. For clinical outcomes (yes or no), the results are presented as ORs and 95% CIs from a comparison of quartiles 2, 3, and 4 with quartile 1 or as predicted probability (0 or 1) adjusted for covariates.

In sensitivity analyses, we additionally adjusted models for the baseline estradiol level due to possible confounding effects, and for total protein level due to protein-bound calcium in blood.

## Results

### Baseline Characteristics

The baseline demographic, reproductive, hormonal and clinical characteristics are shown in **Table 1** (mean ± SD) for a total of 110 women who underwent OS and were grouped by the quartile ranges of the serum folate level and the Ca/Mg ratio. Women aged 30.7±2.8 years were enrolled in the study, and the mean duration of infertility due to tubal (35.5%), male (43.6%), and tubal and male (20.9%) factors was 41.6 ± 28.2 months. In 66.4% of cases, patients had their first IVF/ICSI attempt, while in 33.6%, patients had their second attempt. Basal levels of hormones were as follows: FSH, 6.61±1.87 IU/l; LH, 5.24±3.71 IU/l; AMH, 5.02±3.43 ng/ml; and estradiol, 34.85±12.54 pg/ml. The antral follicle count was 11.28±2.87. Together, these baseline characteristics prove the inclusion criteria for nonadvanced aged, nonobese, normogonadotropic women with tubal and/or male infertility factors and a prognostic normal response to OS that was estimated to be approximately 70% of women seeking infertility treatment (26). Routine basic semen analysis in clinics revealed that 110 male partners in couples has mean semen volume 3.6 ± 1.7 ml, sperm concentration 66.7 ± 54.5 10^6^/ml, motility 55.1 ± 17.4% and progressive motility 40.1 ± 16.7% (**Supplemental Table 1**).

**Table 1:** Baseline characteristics of 110 women by the quartile range of the serum folate level and Ca/Mg ratio^1^.

	Total group	Serum folate, ng/mL		Ratio Ca/Mg	
		Q1 (<10.8)	Q4 (≥33,0)	Q1 (<4.55)	Q4 (≥5.02)
No. of subjects	110	27	26	28	27
Age, years	30.7 ± 2.8^2^	30.5 ± 3.0	31.0 ± 3.1	31.1 ± 2.8	29.9 ± 2.9
BMI, kg/m^2^	22.15 ± 2.87	22.6 ± 3.1	22.3 ± 3.0	21.4 ± 2.6	23.0 ± 3.32
Infertility duration, *months*	41.6 ± 28.2	42.4 ± 29.9	42.0 ± 23.0	38.3 ± 25.8	37.7 ± 26.7
Infertility diagnosis, *n* (%)					
Female tubal factor	39 (35.5)	12 (44.4)	6 (23.1)	10 (35.7%)	11 (40.7%)
Male factor	48 (43.6)	13 (48.1)	12 (46.2)	10 (35.7%)	12 (44.4%)
Tubal + male factors	23 (20.9)	2 (7.4)	8 (30.8)	8 (28.6%)	4 (14.8%)
IVF/ICSI attempt					
1^st^, *n* (%)	73 (66.4)	18 (66.7)	18 (69.2)	22 (78.6%)	15 (55.6%)
2^nd^, *n* (%)	37 (33.6)	9 (33.3)	8 (30.8)	6 (21.4%)	12 (44.4%)
Antral follicle count	11.8 ± 2.87	11.8 ± 3.32	11.7 ± 2.68	11.3 ± 3.22	11.7 ± 3.11
Type of follitropin alpha					
Original, n (%)	55 (50.0%)	15 (55.6%)	12 (46.15%)	11 (39.3%)	16 (59.3%)
Biosimilar, n (%)	55 (50.0%)	12 (44.4%)	14 (53.85%)	17 (60.7%)	11 (40.7%)
FSH dose, IU	1525 ± 260	1492 ± 194	1551 ± 336	1507 ± 238	1501 ± 257
Duration of stimulation, days	9.74 ± 1.05	9.63 ± 0.74	9.89 ± 1.48	9.61 ± 0.99	9.78 ± 1.15
Ovulation trigger					
GnRH-a, n (%)	18 (16.4%)	6 (22.2%)	3 (11.5%)	5 (17.9%)	4 (14.8%)
hCG, n (%)	92 (83.6%)	21 (77.8%)	23 (88.5%)	23 (82.1%)	23 (85.2%)
Embryo transfer day, *n* (%)					
No embryos transferred	12 (10.9)	2 (7.4)	3 (11.5)	5 (17.9%)	3 (11.1%)
Day 3	20 (18.2)	6 (22.2)	3 (11.5)	4 (14.3%)	6 (22.2%)
Day 5	78 (70.9)	19 (70.4)	20 (79.9)	19 (67.8%)	18 (66.7%)
Embryos transferred, *n* (%)					
No embryos transferred	12 (10.9)	2 (7.4)	3 (11.5)	5 (17.8%)	3 (11.1%)
1 embryo	71 (64.5)	20 (74.1)	15 (57.7)	15 (53.6%)	18 (66.7%)
2 embryos	27 (24.5)	5 (18.5)	8 (30.8)	8 (28.6%)	6 (22.2%)
Baseline serum level					
AMH, ng/ml	5.02 ± 3.43	5.10 ± 3.54	5.17 ± 3.72	4.84 ± 3.39	4.54 ± 2.61
FSH, IU/l	6.61 ± 1.87	6.00 ± 2.04	7.08 ± 2.26	6.33 ± 1.79	6.63 ± 2.04
LH, IU/l	5.24 ± 3.71	5.79 ± 5.25	5.15 ± 2.95	4.70 ± 2.29	5.50 ± 3.31
Estradiol, pg/ml	34.85 ± 12.54	37.8 ± 10.9	30.2 ± 14.1*	30.8 ± 13.7	38.1 ± 10.7*
Prolactin, mIU/ml	274.0 ± 109.9	281.7 ± 115.6	263.8 ± 95.9	249.9 ± 93.0	276.6 ± 109.9
APTT, sec	30.9 ± 7.41	31.9 ± 11.3	30.2 ± 8.49	31.5 ± 11.1	30.0 ± 3.63
Testosterone, nmol/l	1.27 ± 0.72	1.31 ± 0.75	1.21 ± 0.96	1.02 ± 0.50	1.39 ± 0.71
Antithrombin, % of activity	106.4 ± 8.62	106.4 ± 10.2	105.9 ± 6.63	104.9 ± 10.8	108.2 ± 7.05
Red blood cells, mln/mkl	4.424 ± 0.348	4.439 ± 0.274	4.448 ± 0.366	4.423 ± 0.310	4.484 ± 0.366
Total protein, g/l	74.1 ± 4.0	73.7 ± 3.4	75.9 ± 3.7	74.2 ± 4.0	73.2 ± 4.9
Vitamin B-12, pg/ml	373.8 ± 167.0	344.5 ± 139.9	403.0 ± 226.6	400.0 ± 200.7	362.3 ± 138.9
Calcium, mg/l	93.7 ± 3.83	92.7 ± 1.45	93.9 ± 3.89	92.6 ± 4.10	95.8 ± 3.41*
Magnesium, mg/l	19.7 ± 1.29	19.7 ± 1.45	20.1 ± 1.24	21.3 ± 0.99	18.6 ± 0.69*

^1^*P < 0.05 for differences across corresponding quartiles. Differences were tested by using a Kruskal-Wallis test for continuous variables and a chi-square test for categorical variables.

^2^Mean ± SD (all such values).

**Figure 3:**
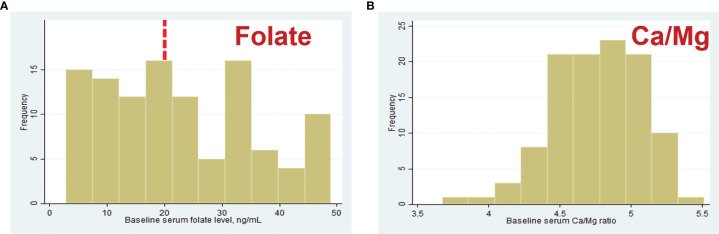
Distribution of baseline serum folate **(A**) and Ca/Mg ratio **(B)** concentrations in 110 women. Red dashed line is represent serum folate level 20 ng/ml considered “elevated” by WHO (27).

The median (IQR) serum concentrations of micronutrients were as follows: folate, 20.55 (10.8, 32.9) ng/ml; Mg, 19.4 (18.7, 20.7) mg/L; Ca, 94 (91.2, 96.4) mg/L; Ca/Mg ratio, 4.78 (4.55, 5.02); and B12, 350 (239, 471) pg/mL. These values were used as thresholds for quartiles for all consequent statistical analyzes. The distributions of the serum folate level and the Ca/Mg ratio are shown in **Figure 3**. Two women in our study had deficient serum folate concentrations, as defined by WHO criteria, below 4 ng/ml (28).

### Comparison of Baseline Characteristics in Groups of Micronutrients

Baseline demographic, reproductive and hormonal characteristics were generally similar across quartiles of the serum folate concentration and the Ca/Mg ratio. Factors considered as inclusion criteria, including age, BMI, infertility cause, baseline FSH and AMH level, were not different, while the serum estradiol level was different. Women in the lowest quartile of serum folate had significantly higher estradiol levels (37.8 ± 10.9 pg/ml) than women in the highest quartile (30.2 ± 14.1 pg/ml), p=0.046, and women in the lowest quartile of the Ca/Mg ratio had significantly lower estradiol levels (30.8 ± 13.7 pg/ml) than women in the highest quartile (38.1 ± 10.7 pg/ml), р=0.02 (**Table 1**). Male partners in the lowest quartile of serum folate had significantly higher sperm concentration (73.5 ± 46.8 10^6^/ml) than men in the highest quartile (45.2 ± 35.6 10^6^/ml), p=0.01 (**Supplemental Table 1**). No differences were found between quartiles of the serum folate level and the Ca/Mg ratio for specific ART parameters, including the number of IVF/ICSI attempts, type of follitropin alpha, duration and dose of FSH stimulation, type of ovulation triggers, and day and number of embryo transfers.

Baseline serum vitamin B-12, calcium and magnesium concentrations were similar across quartiles of serum folate, and serum vitamin B-12 was similar across quartiles of the Ca/Mg ratio. As expected, women in the lowest quartile of the Ca/Mg ratio compared with the highest quartile had significantly lower serum calcium (92.6 ± 4.10 vs. 95.8 ± 3.41 mg/l, p=0.004) and higher magnesium (21.3 ± 0.99 vs. 18.6 ± 0.69 mg/l, p<0.001) (**Table 1**).

### Efficacy of the ART Protocol

In total, 110 women were treated (mean±SD) for 9.74±1.05 days with an average total dose of r-hFSH equal to 1525±260 IU (**Table 1** and **Figure 2**). After fertilization, 2 subjects dropped out from the study due to movement from clinics for family reasons. For 10 patients, embryo transfers were not performed due to the high risk of OHSS for 7 women, no embryos to transfer for 2 women, and no fertilized oocytes for 1 woman. Fresh embryo transfer on days 3 (n=20, 18.2%) and/or 5 (n=78; 70.9%) was performed in 98 women of 110, and the mean number of transferred embryos was 1.27 ± 0.45.

The efficacy of the ART protocol was comparable with data obtained from other trials with different FSH regimens, as summarized in meta-analyses (29, 30) (**Table 2**). The mean number of follicles ≥ 16 mm on the day of trigger administration was 11.7 ± 5.6, the number of oocytes retrieved was 11.9 ± 6.8, the number of matured oocytes (MII stage) was 9.8 ± 5.9, and the number of fertilized oocytes (two-pronuclear zygote, 2PN) was 8.7 ± 6.0. A total of 35.7% of women had biochemical pregnancies, 29.6% had clinical pregnancies, and the take-home baby rate was 27.6%.

**Table 2:** Outcomes of ART in a single fresh GnRH-ant protocol.

Women with OPU (mean ± SD), N = 110	
Follicles ≥16 mm, n	11.7 ± 5.6
Number of oocytes retrieved, n	11.9 ± 6.8
Mature oocytes (MII stage), n	9.8 ± 5.9
Fertilized oocytes (zygotes with 2PN), n	8.7 ± 6.0
**Women with ET, N = 98**	
Biochemical pregnancy (hCG≥25 mIU/ml, 12-17 days after ET), n (%)	35 (35.7)
Clinical pregnancy (ultrasound detection of gestational sac, 10 weeks after ET), n (%)	29 (29.6)
Take-home baby rate, n (%)*	27 (27.6)

*no data about the sex of new-borns.

### Collinearity of Covariates (Inclusion Criteria) and Micronutrients

Using Spearman correlation, we checked the potential collinearity and association of investigated serum micronutrients and covariates, particularly factors that were considered inclusion criteria. Age, the baseline serum AMH level and the number of antral follicles were not correlated with micronutrient levels. BMI and the baseline serum FSH level were positively correlated with the baseline calcium level, with rho=0.24, p=0.01, and rho=0.28, p=0.003, respectively. The serum estradiol concentration was significantly positively correlated with the Ca/Mg ratio (rho=0.27, p=0.005) and significantly negatively correlated with the Mg level (rho=-0.22, p=0.03), while the negative correlation of the baseline estradiol level with the folate level (rho=-0.15, p=0.11) was not significant. Total protein level was significantly positively correlated with both, serum calcium and magnesium level (rho=0.29, p=0.004 and rho=0.24, p=0.019, respectively), but not with Ca/Mg ratio (rho=-0.10, p=0.31).

### Covariates and ART Outcomes

Based on univariate regression analysis, age, male factor of infertility, sperm concentration, baseline levels of total protein, estradiol and LH, type of follitropin alpha, ovulation trigger, and day and number embryo transfer were not associated with biochemical and clinical pregnancies or the live birth rate. A lower age, a higher baseline level of estradiol and the use of GnRH-a as an ovulation trigger (vs hCG) were associated with higher numbers of retrieved, matured and fertilized oocytes, all with p<0.05. Other covariates significantly associated with outcomes that were included in the final multivariate models are presented further.

### Baseline Serum Folate and ART Outcomes

**Figure 4:**
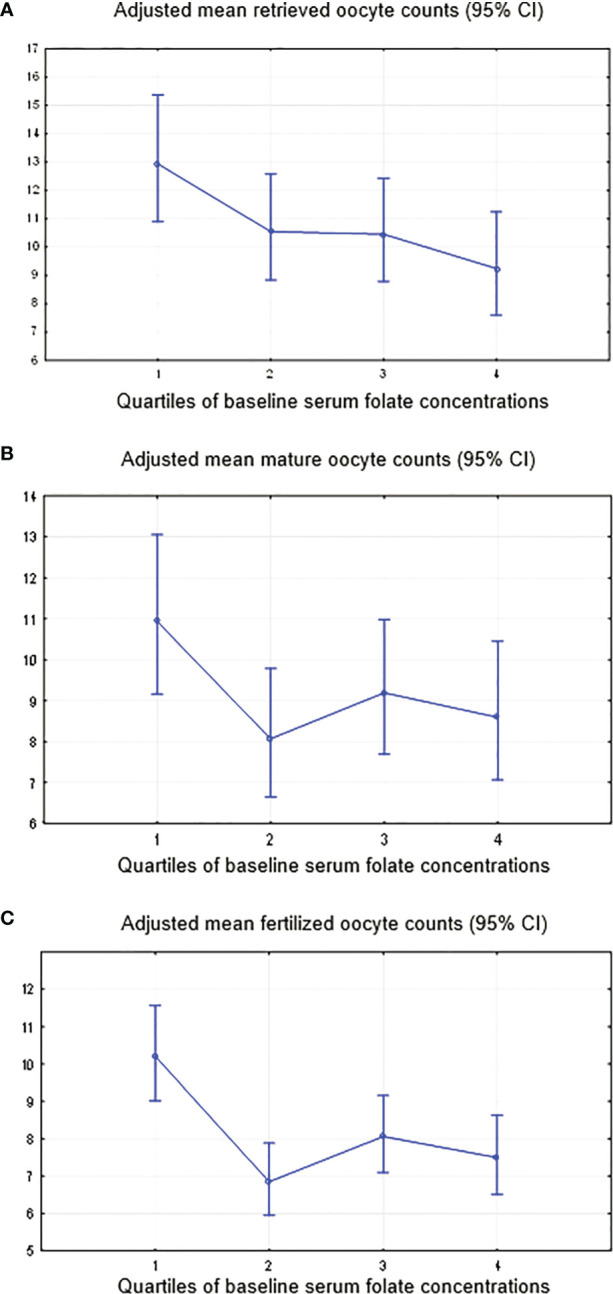
Adjusted mean oocyte counts by the quartiles of the serum folate level. **(A)** Total oocytes yield, adjustment for IVF/ICSI attempts (0; 1) and continuous BMI, baseline serum AMH and APTT, duration of stimulation and dose of FSH. **(B)** Mature oocytes, adjustment for continuous BMI, baseline serum AMH and APTT, duration of stimulation and dose of FSH. **(C)** Fertilized oocytes, adjustment for continuous BMI, baseline serum AMH and APTT, duration of stimulation and dose of FSH.

In the multivariable model, women with higher serum folate concentrations had a significantly lower total number of oocytes retrieved (*P*-trend=0.006), with values of 9.2 (95% CI: 7.6-11.3) in the highest quartile (>=33.0 ng/ml) and 12.9 (95% CI: 10.9-15.4) in the lowest quartile (<10.8 ng/ml). Adjustments were made for the BMI (β-est=-0.04; p=0.01), baseline serum AMH (β-est=0.06; p=0.000), APTT (β-est=-0.02; p=0.01), duration of FSH stimulation (β-est=0.29; p=0.003), FSH dose (β-est=-0.001; p=0.002), and IVF/ICSI attempt (first or second; β-est=-0.12; p=0.01) (**Table 3** and **Figure 4A**). Additional adjustment for the baseline serum estradiol level slightly attenuated the association between the folate level and retrieved oocytes, but it remained significant.

**Table 3:** Associations of serum folate concentrations and early ART outcomes in 110 women.

Quartile (minimum–maximum)	Number of subjects	Total oocytes yield^a^	M2 oocytes^b^	Fertilization (2PN)^b^
		Adjusted mean (95% CI)	Adjusted mean (95% CI)	Adjusted mean (95% CI)
**Serum folate, ng/ml**				
Q1 (2.9 – 10.7)	n=27	12.9 (10.9 – 15.4)	10.9 (9.2 – 13.1)	10.2 (8.36 – 12.6)
Q2 (10.8 – 20.5)	n=28	10.6 (8.8 – 12.6)	8.1 (6.6 – 9.8)	6.8 (5.4 – 8.70)
Q3 (20.6 – 32.9)	n=29	10.5 (8.8 – 12.4)	9.2 (7.7 – 11.0)	8.1 (6.5 – 10.0)
Q4 (≥ 33.0)	n=26	9.2 (7.6 – 11.3)	8.6 (7.1 – 10.5)	7.5 (5.9 – 9.5)
***P-trend*** ^c^		0.006	0.13	0.16
**Ratio Ca (mg/l)/Mg (mg/l)**				
Q1 (3.675 – 4.550)	n=28	10.6 (8.9 – 12.8)	9.1 (7.6 – 10.9)	8.5 (6.8 – 10.5)
Q2 (4.551 – 4.780)	n=27	9.60 (8.0 – 11.6)	7.5 (6.1 – 9.2)*	6.0 (4.6 – 7.7)*
Q3 (4.781 – 5.018)	n=28	11.6 (9.7 – 13.8)	10.0 (8.3 – 11.9)	8.7 (6.9 – 10.8)
Q4 (≥ 5.019)	n=27	11.2 (9.4 – 13.4)	10.3 (8.6 – 12.3)	9.7 (7.8 – 12.0)
***P-trend*** ^c^		0.88	0.16	0.15

Q, quartile.

*P < 0.05 compared with the 1^st^ quartile.

a Analysis was conducted by using generalized linear/nonlinear models with a Poisson distribution for oocyte counts and the log link function for all outcomes with adjustment for IVF/ICSI attempts (0; 1) and continuous BMI, baseline serum AMH and APTT, duration of stimulation and dose of FSH.

b Analyses were conducted by using generalized linear/nonlinear models with a Poisson distribution for oocyte counts and the log link function for all outcomes with adjustment for continuous BMI, baseline serum AMH and APTT, duration of stimulation and dose of FSH.

c Tests for trends across quartiles were conducted by using the nptrend command by STATA (24), and individual predicted values in each model were grouped by quartiles.

Although not significant, the trends of lower oocyte number with higher baseline folate were found for (i) matured oocytes (MII) (*P*-trend=0.13) with adjustments for BMI (β-est=-0.04; p=0.01), baseline serum AMH (β-est=0.06; p=0.000), APTT (β-est=-0.02; p=0.01), duration of FSH stimulation (β-est=0.23; p=0.02) and FSH dose (β-est=-0.001; p=0.02) (**Figure 4B**); and (ii) fertilized oocytes (*P*-trend 0.16), adjusted for BMI (β-est=-0.05; p=0.02), baseline serum AMH (β-est=0.06; p=0.000), APTT (β-est=-0.03; p=0.02), duration of stimulation (β-est=0.28; p=0.02) and FSH dose (β-est=-0.001; p=0.07) (**Figure 4C**). Similar to retrieved oocytes, additional adjustment for the baseline serum estradiol level slightly attenuated the association between the folate level and number of matured or fertilized oocytes.

**Figure 5:**
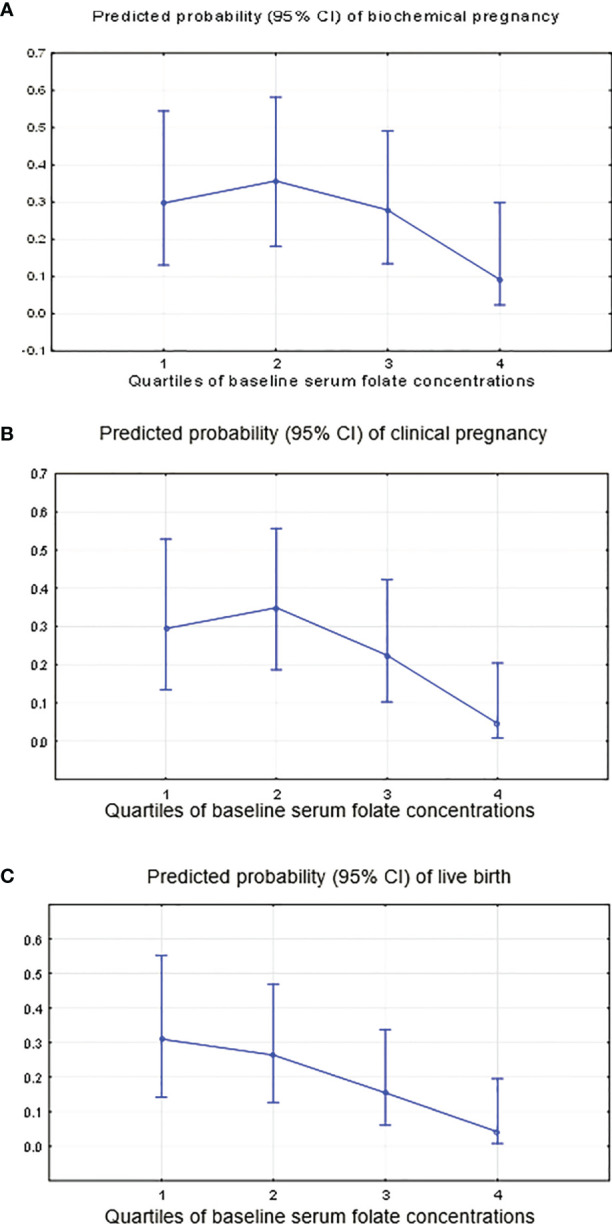
Predicted probability of occurring “no” (0) or “yes” (1) for clinical ART outcomes by the quartiles of the serum folate level. **(A)** Biochemical pregnancy, adjustment for continuous red blood cells, baseline serum FSH and antithrombin. **(B)** Clinical pregnancy, adjustment for continuous red blood cells and baseline serum FSH. **(C)** Live birth, adjustment for continuous red blood cells and baseline serum FSH.

Women with higher serum folate concentrations had significantly lower clinical pregnancy (*P*-trend=0.000) and live birth rates (*P*-trend=0.000) and a tendency towards lower rates of biochemical pregnancy (*P*-trend=0.08) (**Table 4** and **Figures 5A–C**). Specifically, women in the highest quartile of the serum folate level (Q4≥33.0 ng/ml) had a 0.24 (95% CI: 0.04, 1.37) odds ratio (OR) for biochemical pregnancy, with adjustments for red blood cells (OR=10.1; p=0.003), baseline serum FSH (OR=1.6; p=0.002) and antithrombin (OR=0.91; p=0.008) (**Figure 5A**); an OR of 0.12 (95% CI: 0.02, 0.79) for clinical pregnancy with adjustments for red blood cells (OR=5.6; p=0.02*)* and baseline serum FSH (OR=1.4; p=0.02) (**Figure 5B**); and an OR of 0.10 (95% CI: 0.01, 0.70) for live birth, with adjustments for red blood cells (OR=5.8; p=0.03*)* and baseline serum FSH (OR=1.4; p=0.01) compared with women in the lowest quartile (<10.8 ng/ml) (**Figure 5C**). There was no change in the association between the folate level and later ART outcomes when adjusting for the baseline serum estradiol level.

**Table 4:** Associations of serum folate concentrations and clinical outcomes after assisted reproduction in 108 women.

Quartile (minimum–maximum)	Biochemical pregnancy^a^		Clinical pregnancy^b^		Live birth^b^	
	Cases (%)	OR (95% CI)	Cases (%)	OR (95% CI)	Cases (%)	OR (95% CI)
**Serum folate, ng/ml**						
Q1 (2.9 – 10.7)	8/27 (29.6)	1.00 (ref)	8/27 (29.6)	1.00 (ref)	8/27 (29.6)	1.00 (ref)
Q2 (10.8 – 20.5)	11/28 (39.3)	1.31 (0.33, 5.23)	10/28 (35.7)	1.28 (0.35, 4.72)	8/28 (28.6)	0.80 (0.20, 3.10)
Q3 (20.6 – 32.9)	11/29 (37.9)	0.91 (0.23, 3.59)	9/29 (31.0)	0.69 (0.18, 2.62)	7/29 (24.1)	0.40 (0.10, 1.68)
Q4 (≥ 33.0)	5/24 (20.8)	0.24 (0.04, 1.37)*	2/24 (8.33)	0.12 (0.02, 0.79)*	2/24 (8.33)	0.10 (0.01, 0.70)*
***P-trend*** ^c^	0.08		0.000		0.000	
**Ratio Ca (mg/l)/Mg (mg/l)**						
Q1 (3.675 – 4.550)	4/27 (14.8)	1.00 (ref)	3/27 (11.1)	1.00 (ref)	3/27 (11.1)	1.00 (ref)
Q2 (4.551 – 4.780)	7/27 (25.9)	1.92 (0.39, 9.48)	6/27 (22.2)	1.96 (0.39, 9.87)	4/27 (14.8)	1.04 (0.19, 5.70)
Q3 (4.781 – 5.018)	12/27 (44.4)	7.09 (1.45, 34.60)*	9/27 (33.3)	3.14 (0.66, 14.90)	8/27 (29.6)	2.65 (0.54, 12.9)
Q4 (≥ 5.019)	12/27 (44.4)	6.58 (1.31, 33.04)	11/27 (40.7)	4.85 (1.02, 23.08)	10/27 (37.0)	4.07 (0.83, 19.9)
***P-trend*** ^c^	0.000		0.000		0.000	

OR, odds ratio; Q, quartile; ref, reference.

*P ≤ 0.05 compared with the 1^st^ quartile.

a Analysis was conducted by using generalized linear/nonlinear models with a binomial distribution and the log link function with adjustment for continuous red blood cells, baseline serum FSH and antithrombin.

b Analysis was conducted by using generalized linear/nonlinear models with a binomial distribution and the log link function with adjustment for continuous red blood cells and baseline serum FSH.

c Tests for trends across quartiles were conducted by using the nptrend command by STATA (24), and individual predicted values in each model were grouped by quartiles.

### Baseline Ca/Mg Ratio and ART Outcomes

The calcium and magnesium levels (expressed as Ca/Mg ratio) did not have statistically significant impacts on early ART outcomes, including the oocyte yield (p-trend=0.88), number of matured oocytes (p-trend=0.16) and fertilized oocytes (p-trend=0.15), with the same adjustments as for serum folate (**Table 3** and **Supplementary Figures S1A–C**).

**Figure 6:**
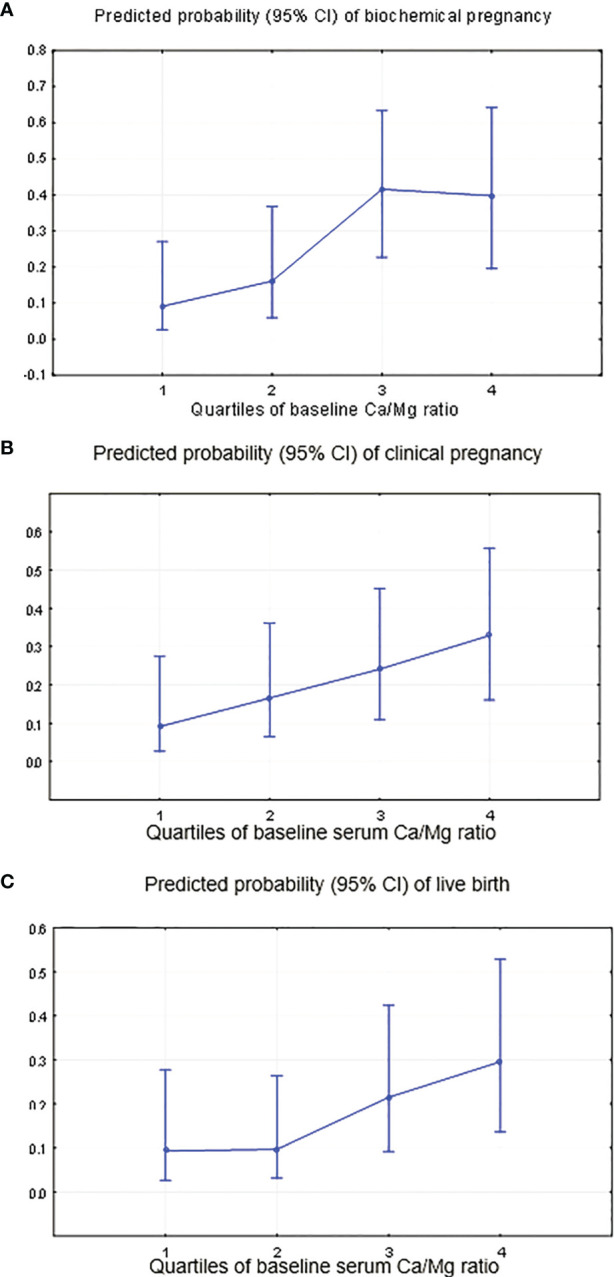
Predicted probability of occurring “no” (0) or “yes” (1) for clinical ART outcomes by the quartiles of the serum Ca/Mg ratio. **(A)** Biochemical pregnancy, adjustment for continuous red blood cells, baseline serum FSH and antithrombin. **(B)** Clinical pregnancy, adjustment for continuous red blood cells and baseline serum FSH. **(C)** Live birth, adjustment for continuous red blood cells and baseline serum FSH.

Women with a higher Ca/Mg ratio had significantly higher biochemical (p-trend=0.000), clinical pregnancy (p-trend=0.000) and live birth rates (p-trend=0.000) (**Table 4** and **Figures 6A–C**). Specifically, women in the highest quartile of the serum Ca/Mg ratio (≥5.02) had a 6.58 (95% CI: 1.31, 33.04) odds ratio (OR) for biochemical pregnancy (**Figure 6A**); an OR of 4.85 (95% CI: 1.02, 23.08) for clinical pregnancy (**Figure 6B**); and an OR of 4.07 (95% CI: 0.83, 19.9) for live birth (**Figure 6C**), with the same adjustments as for serum folate, compared with women in the lowest quartile (<4.55) (**Table 4**). Women in the 3^rd^ quartile of the Ca/Mg ratio (4.78-5.01) had a significant OR of 7.09 (95% CI: 1.45, 34.6) for biochemical pregnancy and a tendency of a higher OR for clinical pregnancy (3.14 (95% CI: 0.66, 14.9)) and life birth (2.65 (95% CI: 0.54, 12.9)). There was no change in the association between the Ca/Mg ratio and ART outcomes when adjusting for the baseline serum estradiol level. There was no change in the regression models when adjusting for total protein.

## Discussion

Adequate vitamin and micronutrient levels are important for oocyte quality, maturation, fertilization, and pregnancy outcomes. However, the amount of biological data accumulated today cannot provide straightforward clinical recommendations for each vitamin and biologically active compound with a focus on human reproduction and ART outcomes (1, 2). In the present work, we investigated the impacts of the baseline serum folate and total Ca and Mg levels (expressed as the Ca/Mg ratio) on ART outcomes in a single fresh GnRH-ant treatment cycle. A total of 110 normal responder women without endocrine and ovarian disturbances (normogonadotropic nonadvanced aged, nonobese with a tubal and/or male infertility factor) were enrolled in the study. They represent one of the main clusters of infertility: fallopian tube blockage or damage occurs in more than 20% of women referred to fertility clinics (31, 32), and up to 40% of cases involve a male factor (33). According to recommendations for normal responder patients (34, 35), the single GnRH-ant protocol using the starting fixed 150 IU daily dose of r-hFSH followed by its adjustment on the basis of the ovarian response was used in this study.

In this study, we found that among nonobese, normogonadotropic women aged 20-35 years, women with higher serum folate levels (≥ 33.0 ng/ml) had significantly lower total numbers of oocytes retrieved and lower odds ratios of 0.24 (0.04, 1.37) for biochemical pregnancy, 0.12 (0.02, 0.79) for clinical pregnancy and 0.10 (0.01, 0.70) for live birth compared with women in the lowest quartile (<10.8 ng/ml).

Recommendations for folate/folic acid intake differ between countries, although the majority of guidelines recommend a healthy diet plus folic acid supplementation at 400 μg/day from the preconception period (up to 3 months) until the end of the first trimester of pregnancy, as issued by the WHO (6). Based on WHO recommendations that first appeared in 1968 addressing the macrocytic anemia concern and that are still active, a deficiency of folate status can be considered with serum folate levels below 3 ng/ml; possible deficiency with levels of 3.0-5.9 ng/ml; the normal range is 6.0-20.0 ng/ml; and “elevated” levels are more than 20 ng/ml (27). According to WHO criteria, the majority of women in the present study (n=83) had normal (n=28, quartile Q2 in the study (10.8-20.5 ng/ml)) and “elevated” (n=55, quartiles Q3 and Q4) serum folate levels, and women with possible deficiency and normal folate levels were included in quartile Q1 (<10.8 ng/ml, **Tables 1**, **3**, **4**).

To date, many studies have tried to assess both the effects of folate intake deficiency and the risk of excessive folic acid intake among women in the periconception period. Inadequate folate intake first leads to a decrease in the serum folate concentration, followed by a decrease in the erythrocyte folate concentration, an increase in the homocysteine concentration, megaloblastic anemia, and changes in the bone marrow and other tissues with rapidly dividing cells (28, 36). In addition, there is strong evidence that folate deficiency during and before pregnancy is associated with low birthweight and neural tube defects (6). Conversely, some studies revealed adverse effects of higher doses or longer consumption of folic acid. Cheng et al, 2019 reported that a longer duration of folic acid supplementation before pregnancy might increase the risk of gestational diabetes mellitus (37). High dosages of folic acid supplements during pregnancy can influence psychomotor development after the first year of life (5). Recently, Field and Stover, 2018, reviewed the safety of folic acid and suggested that additional research is needed to assess the health effects of folic acid supplement use when the current upper limit for folic acid is exceeded (38).

The median (IQR) B12 level was 350 (239, 471) pg/ml in our study, which is higher than the 200 pg/ml considered inadequate by the WHO (28) and higher than the 300 pg/ml recommended for women becoming pregnant to reduce the risk for neural tube defects (39). However, serum B12 concentrations in our study were lower than those measured in women in the EARTH study by Gaskins et al, 2015, who reported positive associations of the serum concentrations of vitamin B12 and folate greater than the median (534 pg/ml for B12 and 20.5 ng/ml for folate) with a higher live birth rate (17). In our study, the baseline vitamin B12 concentration was not associated with any ART outcomes. We did not find folate and B12 correlation (rho=0.15). Furthermore, we did not observe high serum folate concentrations or coexisting B12 deficiency (**Table 1**), which can lead to hyperhomocysteinemia (40).

The biological pathways involved in the action of folates and other micronutrients on the efficacy of ART are still unclear. The possible mechanism that can partially explain the results is related to the interplay between folates, magnesium/calcium levels and estradiol biosynthesis.

As shown in **Table 1**, women in the highest quartile of the serum folate concentration had significantly lower baseline serum estradiol levels than women in the lowest quartile, and a higher serum estradiol level was associated with a higher number of retrieved, matured and fertilized oocytes in univariate models. The steroidogenic potential of ovaries depends on aromatase enzyme complex activity, which converts androgens produced by theca cells to estrogens in granulosa cells (41). This process is modulated through different endocrine, paracrine and autocrine factors and mechanisms. Among them are insulin-like growth factors (IGFs), which affect cell growth and metabolism and are responsible for the release of estradiol from human granulosa cells in a dose-dependent manner (42). Previously, it was found that IGF-1 significantly enhanced the expression of aromatase (CYP19A1) mRNA and caused increased estradiol production (43). IGFs act by binding to specific IGF receptors (types I - II), which are also localized in granulosa cells (42). It has been shown that the IGF-I receptor gene is a novel downstream target for folic acid action (44). Namely, folic acid downregulates IGF receptor type I promoter activity as well as its endogenous mRNA expression and protein levels in a dose-dependent manner (44). The lack of expression of IGF receptors can lead to diminished estradiol biosynthesis in granulosa cells. Previously, it was found that higher folate levels during the IVF programme might modify the ovarian response to OS and have an influence on estradiol levels either directly by modulating follicular FSH responsiveness or through aromatase availability (19). Additionally, a folate-rich diet could have the effect of lowering circulating IGF-1 levels in elderly women (45).

DNA methylation is a key and the most investigated component of epigenetic modifications as well as nutritional epigenomics (46). There is excessive evidence that nutrients, especially folates as methyl donors, may modify DNA methylation as a main physiological process for gene silencing that leads to biological pathway activation/deactivation (46). For example, folate, as a precursor to S-adenosylmethionine, is an essential cofactor for the methylation of catechol estrogens and other derivatives (47) that can also affect estradiol biosynthesis in granulosa. Recently, it has been reported that periconceptional folate levels at both paternal and maternal levels are associated with epigenetic events in gametes, embryos and children (48–51).

Another important and new finding of our study was that a lower baseline serum Ca/Mg ratio was associated with worse clinical ART outcomes in a dose-dependent manner. As shown in **Table 1**, women in the lowest quartile of the Ca/Mg ratio had significantly lower estradiol and calcium levels and higher magnesium levels than women in the highest quartile, while the median (IQR) serum calcium and magnesium concentrations in the present study were 94 (91.2, 96.4) mg/l and 19.4 (18.7, 20.7) mg/l, respectively, and considered normal (52). Investigating five different stages of normal cycling (the menstrual, early follicular, late follicular, ovulatory and luteal phases), Muneyyirci-Delale et al, 1998 presented physiological recurring cycling of ionized Mg and cyclic alterations in the Ca/Mg ratio in the serum of healthy women of reproductive age (13). They reported an increase in the serum Ca/Mg ratio at both the ovulatory and luteal phases when the estradiol level was increasing. The positive significant correlation of the estradiol level and the Ca/Mg ratio in our study may reflect the physiological processes in women described earlier (13).

Based on previous studies, it was shown that calcium and magnesium can modulate estrogen levels and their metabolism. For example, an overall positive association was found between circulating levels of IGF-I and serum calcium (53). Therefore, higher calcium (and a higher Ca/Mg ratio) can be associated with higher IGF-1, which is responsible for the release of estradiol from granulosa cells in a dose-dependent manner (42). Moreover, magnesium is a cofactor of catechol-O-methyl transferase and influences the methylation and excretion of catechol estrogens (54, 55).

We need to address the strengths and limitations of our prospective study. As strengths, aiming to minimize residual confounding, we used a single IVF protocol with fresh embryo transfer in one cycle per normogonadotropic woman. We controlled for more than 30 factors, including a history of infertility (duration and causes, IVF/ICSI attempts), reproductive and applied clinical parameters, baseline hormone levels, biochemical and hemostasis parameters and blood cell counts. Hormone laboratory and clinical staff were blinded to the micronutrient serum levels.

As a limitation, we did not stratify cohort by the male factors including semen quality and their micronutrient status that can influence ART outcomes. However, we collected information about the male factor as the cause of infertility and found that male factor was not associated with ART outcomes in univariate regression models. Additionally as a limitation, we did not estimate the dietary intake and supplement consumption, and we did not measure the serum vitamin D3, iron, and zinc, which can also affect folate, calcium and magnesium levels and ART outcomes. We did not use expensive direct measurement of ionized calcium or albumin-correction of calcium. Instead of this, we used well standardized assessment of total calcium and provided sensitive analysis using adjustment for total protein. We did not found change in association between the Ca/Mg ratio and ART outcomes when adjusting for total protein. We measured hormones in three different accredited clinical laboratories; however, micronutrients were measured in one lab by one batch. Single time-point for lab measurements, the relatively small sample size and selective inclusion criteria are other important limitations. However, “normal responder” (normogonadotropic non-advanced aged, non-obese with tubal and/or male infertility factor) women who were invited in our study were one of most common groups of patients seeking infertility treatment in IVF clinics (26).

It is worth noting that different nutrients are essential during the periconceptional period and pregnancy and can modulate hormone signalling and epigenetic programming of gametes, and it might be associated with early development and offspring health. Special attention should be given to ART cycles that are accompanied by additional epigenetic risks to improve the efficacy of cycles and to provide better health of children. ART cycles can be improved not only by state-of-the-art techniques and treatments but also by guided consumption of “well-known” biologically active substances and nutrients before, during and after ART. Further preconception large-scale studies with known micro- and macronutrient statuses of both parents, including daily doses and serum folate, Ca, Mg, homocysteine and hormone levels, are needed.

## Conclusions

In multivariable models, among nonobese normogonadotropic women aged 20-35 years, a baseline higher serum folate concentration (>33.0 ng/ml) was associated with worse ART outcomes, including biochemical and clinical pregnancies as well as the live birth rate. A baseline lower Ca/Mg ratio (<4.55) was associated with worse clinical ART outcomes in a dose-dependent manner. Our findings might be useful for choosing a safe dosage of folate and other supplementations for both fertile women and women undergoing infertility treatment.

## Data Availability Statement

The original contributions presented in the study are included in the article/**Supplementary Material**. Further inquiries can be directed to the corresponding authors.

## Ethics Statement

The studies involving human participants were reviewed and approved by the Russian Ministry of Health (RCT 754 dated 26.10.16) and the Independent Interdisciplinary Ethics Committee for Ethical Review of Clinical Studies established by Moscow State University of Medicine and Dentistry, Russian Academy of Medical Sciences, Society for Pharmacoeconomics and Outcomes Research, League to support clinical studies and Scientific Center for medical information ‘‘Universimed’’, www.ethicuni.ru (protocol №17 dated 28.10.2016). The patients/participants provided their written informed consent to participate in this study.

## Author Contributions

Data acquisition: ZB, LV, YF, MO, MT, IZ, and VY. Study design, data analysis and interpretation: OS, TU, DB, and MP. Statistical model: OS and TU. Writing original draft: MP. Writing —review and editing: MP and OS. All the authors made substantial contributions to the revision of the article and provided final approval of the version to be published.

## Conflict of Interest

MP is employed by IVFarma LLC; the parental study NCT03088137 was financed by IVFarma LLC. DB served as a medical affairs consultant to Sanofi, Merck and Dr. Reddy’s.

The remaining authors declare that the research was conducted in the absence of any commercial or financial relationships that could be construed as a potential conflict of interest.

## Publisher’s Note

All claims expressed in this article are solely those of the authors and do not necessarily represent those of their affiliated organizations, or those of the publisher, the editors and the reviewers. Any product that may be evaluated in this article, or claim that may be made by its manufacturer, is not guaranteed or endorsed by the publisher.

## Supplementary Material

Supplementary Figure 1 Adjusted mean oocyte counts by the quartiles of the serum Ca/Mg ratio. **(A)** Total oocytes yield, adjustment for IVF/ICSI attempts (0; 1) and continuous BMI, baseline serum AMH and APTT, duration of stimulation and dose of FSH. **(B)** Mature oocytes, adjustment for continuous BMI, baseline serum AMH and APTT, duration of stimulation and dose of FSH. **(C)** Fertilized oocytes, adjustment for continuous BMI, baseline serum AMH and APTT, duration of stimulation and dose of FSH.

## Abbreviations

ART, assisted reproductive technology; OS, ovarian stimulation; IVF, *in vitro* fertilization; r-hFSH, recombinant human follicle-stimulating hormone; IU, International Units; OPU, ultrasound-guided follicular aspiration; AFC, antral follicle count; GnRH-ant, gonadotropin-releasing hormone antagonist; GnRH-a, gonadotropin-releasing hormone agonist; FSH, follicle-stimulating hormone; CI, confidence interval; BMI, body mass index; SC, subcutaneous; OHSS, ovarian hyperstimulation syndrome; ET, embryo transfer; PCOS, polycystic ovary syndrome; hCG, human chorionic gonadotropin; ICSI, intracytoplasmic sperm injection; PN, pronucleus; AMH, anti-mullerian hormone; LH, luteinizing hormone; TSH, thyroid stimulating hormone; ACTH, adrenocorticotropic hormone; GH, growth hormone; APTT, activated partial thromboplastin time; Mg, magnesium; Ca, calcium; IQR, interquartile range; Q, quartile; OR, Odds Ratio; P, probability; LOD, limit of detection; LOQ, limit of quantification; mRNA, messenger ribonucleic acid.
