# Supplementary figures and images for: Association of the Serum Folate and Total Calcium and Magnesium Levels Before Ovarian Stimulation With Outcomes of Fresh In Vitro Fertilization Cycles in Normogonadotropic Women
Source: Front Endocrinol (Lausanne). 2022 Feb 11;13:732731. doi: 10.3389/fendo.2022.732731 (PMC8874277; doi:10.3389/fendo.2022.732731)

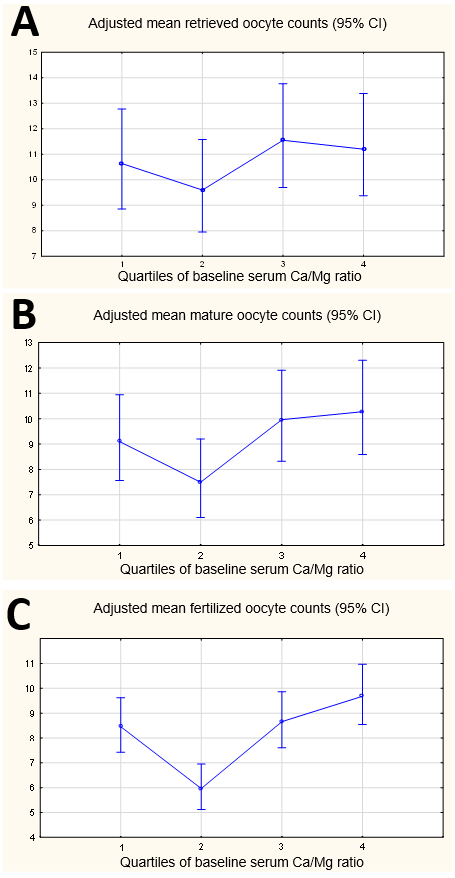

Supplement: Supplementary Figure 1 — Adjusted mean oocyte counts by the quartiles of the serum Ca/Mg ratio. (A) Total oocytes yield, adjustment for IVF/ICSI attempts (0; 1) and continuous BMI, baseline serum AMH and APTT, duration of stimulation and dose of FSH. (B) Mature oocytes, adjustment for continuous BMI, baseline serum AMH and APTT, duration of stimulation and dose of FSH. (C) Fertilized oocytes, adjustment for continuous BMI, baseline serum AMH and APTT, duration of stimulation and dose of FSH. [file Image_1.tif]
