# Supplementary material for: Association of the Serum Folate and Total Calcium and Magnesium Levels Before Ovarian Stimulation With Outcomes of Fresh In Vitro Fertilization Cycles in Normogonadotropic Women
Source: Front Endocrinol (Lausanne). 2022 Feb 11;13:732731. doi: 10.3389/fendo.2022.732731 (PMC8874277; doi:10.3389/fendo.2022.732731)
Supplement: Supplementary file 2 [file Table_1.docx]

**Supplemental Table 1.** Basic semen parameters among 110 male partners in couples by the quartile range of the female serum folate level and Ca/Mg ratio[^1^](https://www.ncbi.nlm.nih.gov/pmc/articles/PMC4588741/table/tbl1/?report=objectonly#tblfn1)

|  | **Total group** | **Serum folate, ng/mL** | | **Ratio Ca/Mg** | |
| --- | --- | --- | --- | --- | --- |
|  |  | **Q1 (<10.8)** | **Q4 (≥33,0)** | **Q1 (<4.55)** | **Q4 (≥5.02)** |
| No. of subjects | 110 | 27 | 26 | 28 | 27 |
| Semen volume, ml | 3.6±1.7^2^ | 3.4±1.6 | 3.8±1.8 | 3.9±2.1 | 3.2±1.3 |
| Sperm concentration, 10^6^/ml | 66.7±54.5 | 73.5±46.8 | 45.2±35.6* | 52.1±40.7 | 88.5±76.7 |
| Motility, % | 55.1±17.4 | 56.4±16.6 | 52.3±19.2 | 57.1±20.2 | 57.0±16.0 |
| Progressive motility, % | 40.1±16.7 | 37.4±16.2 | 36.1±20.4 | 36.3±17.6 | 45.0±16.3 |

^1^**P* < 0.05 for differences across corresponding quartiles. Differences were tested by using a Kruskal-Wallis test.

^2^Mean ± SD (all such values).
